# Extracellular vesicles-associated tRNA-derived fragments: Emerging insights into cancer progression and clinical application potential

**DOI:** 10.1016/j.gendis.2025.101682

**Published:** 2025-05-12

**Authors:** Yanru Pan, Bo Zhang, Zhe Li, Kefeng Hu

**Affiliations:** a Department of Gastroenterology, The First Affiliated Hospital of Ningbo University, Ningbo, Zhejiang 315010, China; b Health Science Center, Ningbo University, Ningbo, Zhejiang 315211, China

**Keywords:** Cancer biomarkers, Clinical application, Exosome-associated tRFs, Personalized cancer therapy, Tumor progression

## Abstract

Exosome-associated tRNA-derived fragments (tRFs) play crucial roles in cancer progression, influencing cell proliferation, metastasis, immune evasion, and drug resistance. Due to their stability and specific expression in bodily fluids, exosomal tRFs hold great promise as non-invasive biomarkers for early cancer diagnosis, treatment monitoring, and prognosis evaluation. Additionally, tRFs present opportunities as therapeutic targets, with potential to modulate oncogenic processes, such as controlling gene expression and modulating cellular signaling pathways. This review explores the diverse functions of exosome-associated tRFs in tumor biology, highlighting their potential for clinical applications, including their use as diagnostic tools and their role in therapy. It also addresses the challenges that remain in standardizing detection methods and validating their efficacy in clinical settings, such as variability in isolation techniques and the need for large-scale studies. Advanced technologies and further research will be key to unlocking their potential in personalized cancer therapy, ultimately aiming to integrate tRFs into routine clinical practice for better patient outcomes.

## Introduction

Exosomes, the smallest subtype of extracellular vesicles (EVs), are critical mediators of intercellular communication within the tumor microenvironment (TME). They modulate interactions between tumor cells, immune cells, stromal components, and endothelial cells, playing an integral role in shaping tumor progression and influencing therapeutic outcomes.^1^, ^2^, ^3^ Exosomes are carriers of a diverse range of bioactive molecules, including proteins, nucleic acids, lipids, and metabolites, which impact processes such as immune modulation, tumor metabolism, and the development of drug resistance.^1^^,^^4^^,^^5^ Recent advances in targeting components of the TME, particularly through immune checkpoint inhibitors, have transformed the landscape of cancer therapy.^6^^,^^7^ Notably, exosomes have emerged as key players in facilitating intercellular communication between donor and recipient cells, contributing to immune evasion, angiogenesis, and metastasis.^8^

Increasing evidence suggests that non-coding RNAs, especially tRNA-derived fragments (tRFs), play important roles in a variety of biological processes, especially epigenetic regulation, gene expression regulation, and immune system function. Epigenetic regulation involves mechanisms such as DNA methylation, histone modifications, chromatin remodeling and ncRNA regulation, which are critical for regulating gene expression and the development and function of the immune system.^9^ For example, histone modifications serve as an essential means of regulating gene expression by altering chromatin structure, thereby influencing the accessibility of genes and modulating the development and activation of immune cells.^10^ In addition, tRFs can participate in RNA interference as miRNA-like molecules by binding to AGO proteins, thereby regulating gene expression.^11^ tRFs can also directly bind to mRNA, inhibiting its translation or promoting its degradation, thereby affecting protein synthesis.^12^ In the immune system, tRFs also play crucial roles, tRFs contribute to immune activation by impacting both innate and adaptive immune responses. The innate immune system relies on complex signaling pathways to recognize pathogens and initiate immune responses, and these pathways are subject to epigenetic regulation at multiple levels.^13^ For instance, epigenetic mechanisms like histone modifications and DNA methylation dynamically regulate the gene expression of immune cells, thereby shaping their ability to respond to pathogens.^14^ In cancer biology, these small RNA molecules, produced through the cleavage of mature or precursor tRNAs, have been implicated in regulating a variety of cellular processes relevant to tumor growth, metastasis, and immune response. For example, tRF-5′-Gly-CCC can inhibit the proliferation of tumor cells by inhibiting the expression of cyclin D1.^15^ tRF-3′-Leu-CAG promotes tumor cell invasion and metastasis by regulating the expression of Epithelial–Mesenchymal Transition (EMT) related transcription factors such as ZEB1 and Snail.^16^ tRF-5′-Val-CAC can enhance T cell activity in the tumor microenvironment by inhibiting the expression of PD-L1, thereby inhibiting tumor growth.^17^ tRF-5′-Glu-TTC can inhibit tumor angiogenesis by inhibiting the expression of VEGF.^18^ In addition, hypoxic conditions within the TME can stimulate the production of tRFs, which destabilize tumor suppressor genes, as observed in breast cancer. Specific tRFs, such as those interacting with PIWI proteins in lung cancer and chronic lymphocytic leukemia, have also been identified as key regulators of tumor progression.^19^ These findings indicate that tRFs not only play pivotal roles in cancer-related molecular mechanisms but also hold potential as biomarkers for predicting patient prognosis and evaluating therapeutic efficacy.

One of the most promising aspects of tRFs is their stability and ability to be packaged into exosomes, facilitating their transport and protection from extracellular nucleases. This “exosomal tRFs” packaging not only increases their stability but also allows them to be secreted into bodily fluids, such as blood and urine, where they can serve as accessible biomarkers for cancer diagnosis and prognosis. For example, plasma exosomal small RNA tRF-Lys-CTT-049 has been identified as a potential early diagnostic marker in non-small cell lung cancer.^20^ Moreover, exosomal tRFs play a functional role in modulating the TME by influencing immune responses, promoting drug resistance, and facilitating cancer metastasis.^11^^,^^20^

Despite the promising potential of exosomal tRFs, several challenges remain. While their role as biomarkers is well-supported by existing studies, the precise molecular mechanisms through which exosomal tRFs contribute to cancer progression are still not fully understood. Additionally, their dysregulated expression across different types of cancers suggests a complex and context-dependent role that warrants further investigation.^11^^,^^20^ Given their unique stability and resistance to degradation, exosomal tRFs represent a promising avenue for the development of liquid biopsy techniques and targeted cancer therapies. However, further research is needed to unravel the mechanisms by which exosomal tRFs contribute to tumorigenesis and how they can be effectively targeted in clinical settings.

In this review, we will explore the multifaceted roles of exosome-derived tRFs within the TME, with a particular focus on their involvement in cancer-related processes, including immune modulation, drug resistance, proliferation, and metastasis. Additionally, we will highlight recent advances in utilizing exosomal tRFs as diagnostic biomarkers and therapeutic targets, offering insights into their potential application in cancer diagnostics, prognosis, and treatment.

## Exosomes and exosome-associated tRFs

### Exosome biogenesis

Exosomes are nanoscale membrane vesicles released into the extracellular milieu, composed of transmembrane lipid bilayer and various substances, containing a variety of essential biomolecules, such as proteins, lipids, functional RNA species, and other bioactive substances that play pivotal roles in various cellular functions.^21^ These vesicles are found in various bodily fluids, such as blood, urine, pleural and peritoneal fluid, cerebrospinal fluid, and saliva.^22^ Exosome biogenesis involves a complex, multi-step process, including the inward budding of the plasma membrane and the formation of multivesicular bodies (MVBs). This process can be broadly divided into four key stages: cargo sorting into MVBs, MVB formation, MVB transport, and MVB fusion with the plasma membrane.^23^ Exosome biogenesis involves a complex, multi-step process, including the inward budding of the plasma membrane and the formation of MVBs. This process can be broadly divided into four key stages: cargo sorting into MVBs, MVB formation, MVB transport, and MVB fusion with the plasma membrane.^23^

**Figure 1:**
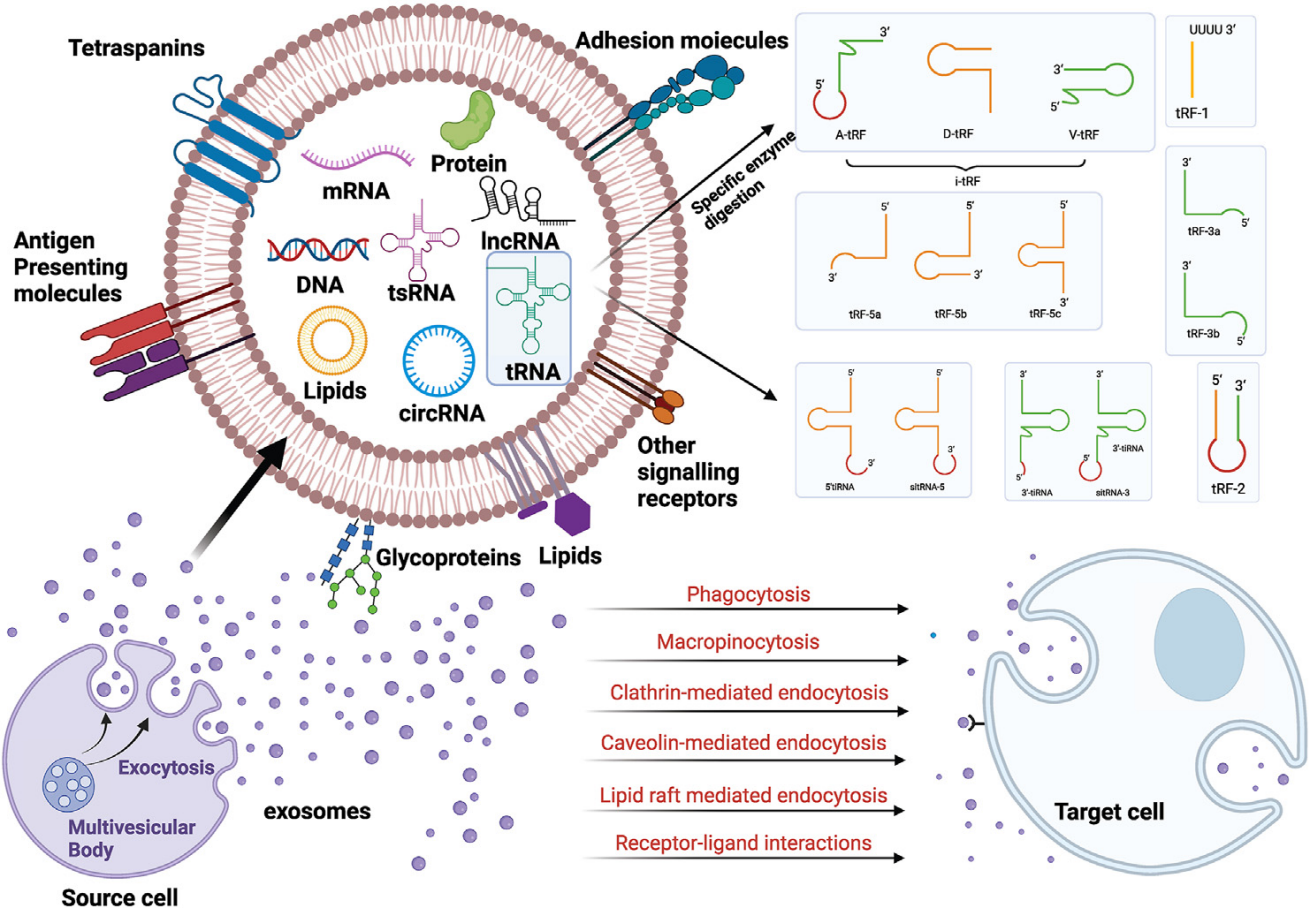
Schematic Representation of Exosome-Mediated Transport and Functions of tRFs. Exosomes are released through exocytosis and contain proteins, lipids, DNA, RNA, and small RNAs like tRFs and tiRNAs. These vesicles have lipid bilayers enriched with transmembrane proteins, glycoproteins, adhesion molecules, and signaling receptors. tRFs are generated by enzymatic cleavage of tRNA and are classified into i-tRF, tRF-1, tRF-2, tRF-3, and tRF-5, while tiRNAs include 5′ tiRNA and 3′ tiRNA. Exosomes interact with target cells via mechanisms like endocytosis, phagocytosis, and receptor–ligand interactions.

Once formed, exosomes deliver their cargo, including proteins and nucleic acids, to recipient cells either through direct fusion with the plasma membrane or via various endocytosis pathways. These pathways include phagocytosis, macropinocytosis, clathrin-dependent endocytosis, caveolin-mediated endocytosis, lipid raft-mediated endocytosis, and receptor–ligand interactions.^8^ Notably, recent studies have shown that the RNA composition of exosomes, including microRNAs (miRNAs), differs from that of their parental cells, likely due to selective packaging during exosome biogenesis,^24^ The process of exosome formation, which involves the inward budding of MVBs, may selectively incorporate specific types of RNA molecules.^25^^,^^26^ Furthermore, exosome release and uptake processes, including interactions with recipient cells such as endothelial cells, may influence RNA composition by selectively releasing or degrading RNA molecules during transport.^27^ This highlights the dynamic and selective nature of exosome biogenesis and content packaging, which has significant implications for intercellular communication in various physiological and pathological processes (Fig. 1).

### Exosome-tRF interrelationships

Exosomes, as small extracellular vesicles with diameters ranging from 40 to 160 nm, are known to transport a diverse range of biologically active molecules, including proteins, lipids, mRNAs, and small RNAs.^28^ In recent years, attention has focused on the discovery that exosomes also carry a novel class of non-coding RNAs known as tRFs.^29^ tRFs are small RNA molecules generated by the specific cleavage of mature tRNAs or their precursor molecules. Based on their cleavage site, tRFs are typically classified into 5′-tRFs, 3′-tRFs, and tRNA halves.^30^ These tRFs perform various biological functions in recipient cells, particularly in the regulation of gene expression through binding to mRNAs or interacting with proteins to modulate key signaling pathways.^9^ Through these mechanisms, tRFs influence a range of cellular processes, including growth, differentiation, stress responses, and metabolic regulation (Fig. 1).

The presence of tRFs in exosomes has attracted considerable attention in cancer research, where they are increasingly recognized for their role in tumorigenesis. Exosomal tRFs facilitate the exchange of molecular information between tumor cells and the surrounding microenvironment, influencing critical processes such as tumor initiation, progression, invasion, and metastasis. For example, specific tRFs have been shown to aid tumor cells in evading immune surveillance by promoting angiogenesis and suppressing immune cell activity.^31^ Moreover, exosomal tRFs contribute to the development of drug resistance, further complicating cancer treatment. Given their stability, abundance, and cancer type-specific expression, exosomal tRFs hold great promise as biomarkers for cancer diagnosis, early detection, and prognosis evaluation.^32^, ^33^, ^34^ These attributes make tRFs valuable tools for non-invasive cancer diagnostics, particularly through the development of liquid biopsy techniques.

In addition to their role as biomarkers, the functional relevance of exosomal tRFs in cancer is becoming increasingly clear. By modulating key signaling pathways in recipient cells, tRFs can influence the behavior of both tumor and stromal cells, contributing to the overall dynamic of the TME. However, despite growing evidence of their importance, much remains to be understood about the specific mechanisms through which exosomal tRFs drive tumor progression and their potential therapeutic applications. Ongoing research is needed to elucidate the full range of functions tRFs may serve within exosomes, as well as to explore how these small RNA molecules can be leveraged for therapeutic targeting in cancer.

## The role of exosome-associated tRF in tumor progression

Increasing evidence suggests that tRFs associated with exosomes exhibit abnormal expression in cancer cells and tissues. These exosome-associated tRFs play pivotal roles in tumor progression by influencing various mechanisms, including modulation of the TME, enhancement of drug resistance, and regulation of tumor cell proliferation and metastasis.

### Regulating the tumor microenvironment

Exosome-associated tRFs significantly contribute to the regulation of the TME, which is composed of tumor cells and their surrounding stroma, blood vessels, immune cells, fibroblasts, and other cellular components. The intricate interactions between these components shape tumor growth, progression, and metastasis.^35^ Exosomal tRFs exert their influence on the TME through multiple mechanisms, promoting malignant behavior.

A study on pancreatic ductal adenocarcinoma (PDAC) revealed that exosomal tRF-GluCTC-0005 binds to the 3′ untranslated region of WDR1 mRNA in hepatic stellate cells (HSCs), enhancing its stability. WDR1, through its interaction with YAP protein, suppresses YAP phosphorylation and promotes its transcriptional activity. This cascade, triggered by tRF-GluCTC-0005, facilitates the recruitment of myeloid-derived suppressor cells (MDSCs) into the liver, creating an immunosuppressive microenvironment dominated by polymorphonuclear neutrophils (PMNs), which promotes hepatic metastasis of PDAC.^36^ This study highlights how exosomal tRFs can drive tumor progression by shaping the TME and mediating immune evasion.

Additionally, exosome-associated tRFs are implicated in promoting the polarization of macrophages toward an M2-like phenotype, which is associated with tumor-promoting functions. For instance, tRF-TSNA-14783 has been shown to promote M2 polarization by increasing the expression of markers such as TGF-β, IL-10, and CD206, while reducing the expression of M1 macrophage markers such as IL-1 and NOS2.^37^ Moreover, tRFs can attenuate the production of pro-inflammatory cytokines by inhibiting the NF-κB signaling pathway, establishing an anti-inflammatory environment conducive to M2 polarization.^37^ Moreover, tRFs can attenuate the production of pro-inflammatory cytokines by inhibiting the NF-κB signaling pathway, establishing an anti-inflammatory environment conducive to M2 polarization.^38^ In colorectal cancer, tRF-3022b, an exosomal tRF, binds to galectin-1 (LGALS1) and macrophage migration inhibitory factor (MIF), modulating MIF levels to reduce M2 macrophage polarization, which impacts tumor growth.^39^

**Figure 2:**
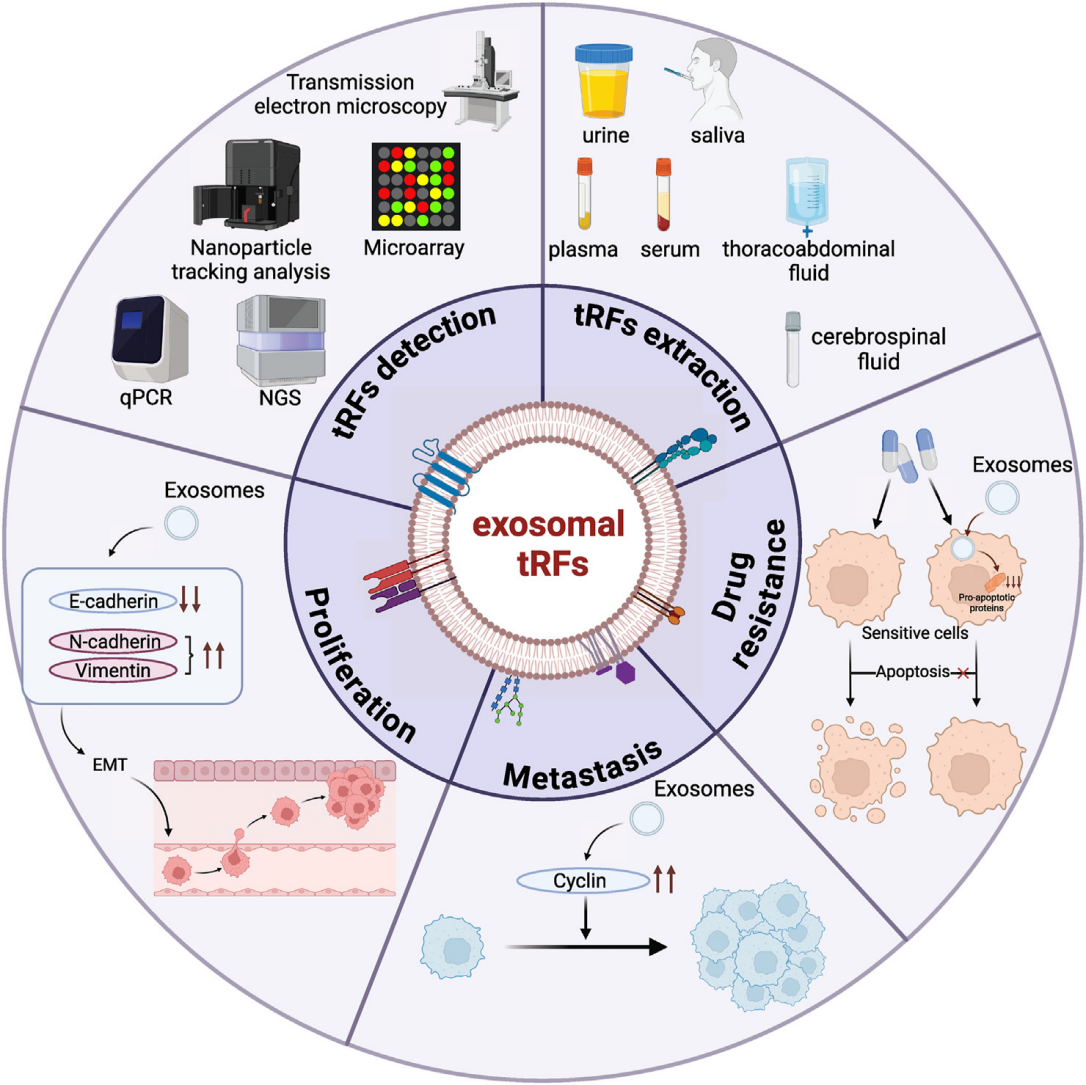
Functional Roles and Detection of Exosomal tRFs in Cancer Progression. Exosomal tRFs are detectable in various body fluids like urine, saliva, serum, plasma, and cerebrospinal fluid using methods such as transmission electron microscopy (TEM), nanoparticle trackinhg analysis (NTA), microarray, qPCR, and next-generation sequencing (NGS). They promote drug resistance by reducing pro-apoptotic proteins, enhance metastasis through EMT by modulating E-cadherin and N-cadherin levels, and support tumor proliferation by upregulating cyclin expression.

In summary, exosome-associated tRFs regulate the TME through various mechanisms that support tumor growth and metastasis, providing new potential targets for cancer therapy (Fig. 2).

### Drug resistance

Exosome-mediated intercellular communication is increasingly recognized as a novel mechanism contributing to drug resistance in cancer. Exosome-associated tRFs play a significant role in the development of resistance to chemotherapy and targeted therapies. Studies have demonstrated that certain exosomal tRFs contribute to drug resistance by modulating apoptosis and survival pathways.

For example, in breast cancer (BC), tRF-16-K8J7K1B has been found to be upregulated in tamoxifen-resistant cells compared to tamoxifen-sensitive cells. High levels of tRF-16-K8J7K1B are associated with shorter disease-free survival in hormone receptor-positive (HR+) BC patients. Mechanistically, exosomal tRF-16-K8J7K1B confers tamoxifen resistance by reducing drug-induced apoptosis. It achieves this by downregulating apoptosis-related proteins such as caspase-3 and poly (ADP-ribose) polymerase (PARP) through targeting TNF-related apoptosis-inducing ligand (TRAIL) receptors.^40^ Furthermore, extracellular tRF-16-K8J7K1B is packaged into exosomes and transferred to recipient cells, where it induces tamoxifen resistance. *In vivo* experiments have shown that inhibiting exosomal tRF-16-K8J7K1B enhances BC cell sensitivity to tamoxifen, suggesting its potential as a therapeutic target to overcome drug resistance in HR + BC patients.^40^

Overall, exosomal tRFs contribute to chemotherapy and radiotherapy resistance in tumor cells and can transmit this resistance to sensitive cells. Targeting exosomal tRFs may provide a novel approach to overcoming drug resistance in cancer treatment (Fig. 2).

### Regulation of tumor cell proliferation and metastasis

Exosome-associated tRFs play a crucial role in regulating tumor cell proliferation and metastasis by modulating gene expression and signaling pathways. These tRFs are transferred to tumor cells via exosomes, where they alter the expression of genes involved in cell cycle progression and EMT, thus promoting tumor progression.^29^ For instance, certain tRFs upregulate the expression of cell cycle-related genes, enhancing tumor cell proliferation.^30^^,^^39^ Additionally, tRFs can promote EMT, a process critical for metastasis, by downregulating epithelial markers such as E-cadherin and upregulating mesenchymal markers such as N-cadherin and vimentin, thereby enhancing tumor cell motility and invasiveness.^41^

In pancreatic cancer, exosomal tRF-19-PNR8YPJZ derived from pluripotent stem cells (PSC) targets AXIN2 in pancreatic cancer cells, reducing its expression and thereby activating the Wnt signaling pathway, which promotes tumor proliferation and metastasis.^42^ Similarly, in endometrial cancer, serum exosome-derived tRF-20-S998LO9D has been shown to inhibit cell proliferation, migration, and invasion, while promoting apoptosis. Knockdown of tRF-20-S998LO9D confirmed its tumor-suppressive role.^43^ Moreover, exosomes derived from lipoma tissue (Exo-LT) have been found to enhance the proliferation and migration of adipose-derived stem cells (ADSCs) compared to exosomes from normal adipose tissue, suggesting that exosomal tRFs may play a role in supporting tumor growth by modulating surrounding stromal cells.^44^

In conclusion, exosome-associated tRFs promote tumor proliferation and metastasis by regulating key processes such as cell cycle progression and EMT. Their pivotal role in tumor progression renders them promising targets for therapeutic intervention, and further research into their mechanisms could offer new strategies for cancer treatment (Fig. 2).

## Clinical significance of exosome-associated tRFs

Exosome-associated tRFs have demonstrated considerable potential for clinical applications. Firstly, tRFs exhibit remarkable stability in bodily fluids and display distinct expression patterns across different cancer types, making them ideal biomarkers for early cancer diagnosis and monitoring through liquid biopsy technologies.^45^ Secondly, the expression levels of tRFs can serve as indicators of tumor response to treatment. By assessing changes in exosomal tRF levels before and after therapy, clinicians can evaluate therapeutic efficacy and predict patient prognosis.^46^ Moreover, the diverse roles of tRFs in tumor progression render them promising therapeutic targets. Specific molecules can be designed to inhibit or enhance the function of particular tRFs, thereby intervening in tumor cell proliferation, metastasis, and drug resistance.^9^^,^^29^^,^^35^, ^36^, ^37^, ^38^, ^39^, ^40^, ^41^, ^42^, ^43^, ^44^ Finally, the specific expression patterns of tRFs enable the development of personalized treatment strategies. By analyzing the profile of exosomal tRFs in patient bodily fluids, tailored therapeutic plans can be formulated, optimizing treatment efficacy while minimizing adverse reactions. Collectively, exosome-associated tRFs hold significant clinical potential for early cancer detection, treatment monitoring, identification of therapeutic targets, and the development of personalized treatment strategies.

### Exosome-associated tRFs as clinical biomarkers

**Figure 3:**
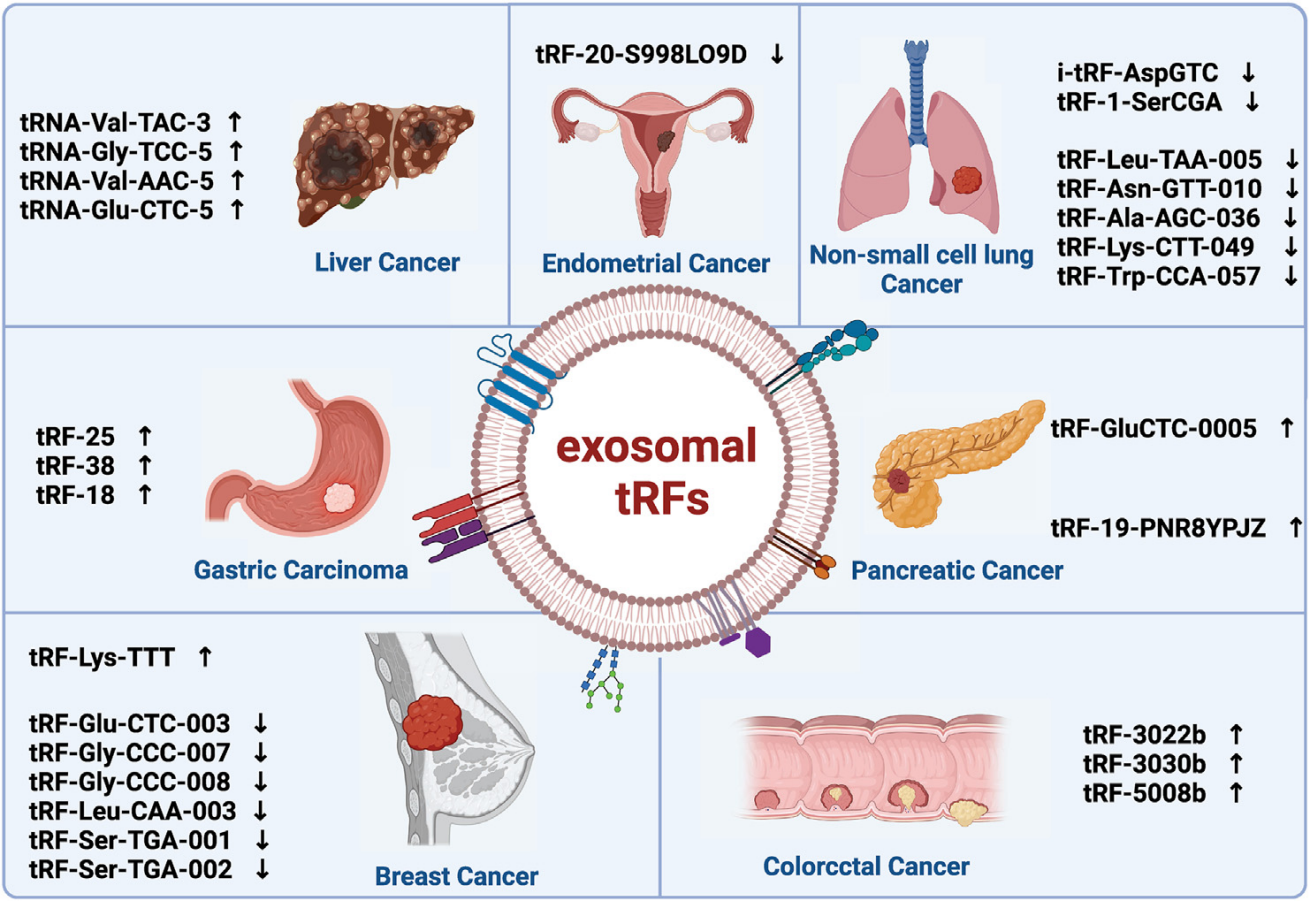
Differential Expression of Exosomal tRFs Across Various Cancer Types. In various cancers, specific exosomal tRFs exhibit differential expression. Liver cancer (HCC) shows upregulation of tRNA-Val-TAC-3, tRNA-Gly-TCC-5, tRNA-Val-AAC-5, and tRNA-Glu-CTC-5. Gastric cancer (GC) has elevated tRF-25, tRF-38, and tRF-18. Breast cancer (BC) shows upregulation of tRF-Lys-TTT and downregulation of multiple tRFs like tRF-Glu-CTC-003. Colorectal cancer (CRC) displays increased levels of tRF-3022b, tRF-3030b, and tRF-5008b. Pancreatic cancer (PDAC) has elevated tRF-GluCTC-0005. Non-small cell lung cancer (NSCLC) and endometrial cancer (EC) show downregulation of various tRFs.

Exosome-associated tRFs have significant potential as clinical biomarkers for cancer. Due to the protective nature of exosomes, tRFs are highly stable in body fluids and are well-suited for non-invasive liquid biopsy. Moreover, the specific expression patterns of tRFs in different cancer types enhance their utility for early diagnosis and for distinguishing between cancer subtypes. By monitoring dynamic changes in exosomal tRF levels in bodily fluids, clinicians can perform real-time assessments of treatment efficacy, and predict recurrence and metastasis. This information is invaluable for optimizing treatment strategies and improving patient outcomes (Fig. 3 and Table 1).

**Table 1:** Overview of Exosome-Associated tRFs in Various Cancer Types: Expression, Sources, and Clinical Relevance.

Exosomal tRF	Type of disease	Source of exosome	Expression	Case number	AUC	Sensitivity	Specificity	References	Clinical relevance
tRNA-Val-TAC-3	Liver cancer	Cell culture medium and plasma	↑	N/A	N/A	N/A	N/A	47	N/A
tRNA-Gly-TCC-5			↑	N/A	N/A	N/A	N/A		N/A
tRNA-Val-AAC-5			↑	N/A	N/A	N/A	N/A		N/A
tRNA-Glu-CTC-5			↑	N/A	N/A	N/A	N/A		N/A
tRF-25	Gastric carcinoma	Plasma	↑	100	N/A	N/A	N/A	34	N/A
tRF-38			↑		N/A	N/A	N/A		N/A
tRF-18			↑		N/A	N/A	N/A		N/A
tRF-Lys-TTT	Breast cancer	Serum and cell culture medium	↑	150	combine: 0.920	74.4% in training cohort 71.8% in validation cohort	80.6% in training cohort 72.2% in validation cohort	48	CEA, CA15-3
tRF-Glu-CTC-003	Breast cancer	Plasma	↓	316	0.684	N/A	N/A	18	Worse disease-free survival and overall survival
tRF-Gly–CCC–007			↓		0.758	N/A	N/A		N/A
tRF-Gly–CCC–008			↓		0.630	N/A	N/A		N/A
tRF-Leu-CAA-003			↓		0.772	N/A	N/A		N/A
tRF-Ser-TGA-001			↓		0.740	N/A	N/A		N/A
tRF-Ser-TGA-002			↓		0.739	N/A	N/A		N/A
Combine					0.844	87.5%	68.8%		N/A
tRF-3022b	Colorectal cancer	Tissues and plasma	↑	161	tissues: 0.8266 plasma: 0.7684	N/A	N/A	39	N/A
tRF-3030b			↑		tissues: 0.8066 plasma: 0.7485	N/A	N/A		N/A
tRF-5008b			↑		tissues: 0.8329 plasma: 0.6921	N/A	N/A		N/A
tRF-GluCTC-0005	Pancreatic cancer	Serum	↑	234	0.783	N/A	N/A	36	Liver metastasis CA19-9
tRF-19-PNR8YPJZ	Pancreatic cancer	PSCs culture medium	↑	160	0.8388	N/A	N/A	42	Lymph node invasion Metastasis Perineural invasion Advanced clinical stage
i-tRF-AspGTC	Non-small cell lung cancer	Serum	↓	443	Predictive diagnostic: 0.690 Early diagnostic: 0.656	N/A	N/A	50	CEA, CYFRA21-1
tRF-1-SerCGA			↓		Predictive diagnostic: 0.680 Early diagnostic: 0.688	N/A	N/A		
Combine					Predictive diagnostic: 0.702 Early diagnostic: 0.683	N/A	N/A		
tRF-Leu-TAA-005	Non-small cell lung cancer	Plasma	↓	542	0.7420	68.4%	72.4%	20	N/A
tRF-Asn-GTT-010			↓		0.7574	80.7%	59.6%		N/A
tRF-Ala-AGC-036			↓		0.7371	66.4%	71.2%		Tumor stage
tRF-Lys-CTT-049			↓		0.7066	71.3%	63.6%		N/A
tRF-Trp-CCA-057			↓		0.6677	57.0%	71.6%		N/A
Combine					0.7608	80.7%	62.0%		N/A
tRF-20-S998LO9D	Endometrial carcinoma	Serum	↓	80	0.768	N/A	N/A	43	N/A

### Exosomal tRFs in liver cancer

Zhu et al. demonstrated the widespread presence of tRFs in exosomes from liver cancer cell culture media and plasma. In hepatocellular carcinoma (HCC) cell culture media, 5′-tRFs were the most abundant (90%), followed by 3′-tRFs (9%) and i-tRFs (1%). Plasma exosomal tRFs were significantly elevated in HCC patients compared with healthy controls, with 5′-tRFs being the predominant subtype. Notably, four specific plasma exosomal tRFs—tRNA-Val-TAC-3, tRNA-Gly-TCC-5, tRNA-Val-AAC-5, and tRNA-Glu-CTC-5—were significantly upregulated in HCC patients, indicating their potential as biomarkers for HCC diagnosis.^47^

### Exosomal tRFs in gastric cancer

Lin et al. found that plasma exosomal tRF-25, tRF-38, and tRF-18 were significantly upregulated in gastric cancer (GC) patients compared to healthy controls. This was based on plasma samples from 50 healthy individuals and 50 GC patients. These findings suggest the potential of these tRFs as diagnostic and prognostic biomarkers for GC.^34^

### Exosomal tRFs in breast cancer

Koi et al. discovered that miR-23a-3p, miR-21-5p isomiR, and tRF-Lys-TTT were significantly upregulated in BC samples. During the screening phase, the sensitivity of these three small RNAs for BC diagnosis was 76.9%, 69.2%, and 74.4%, respectively, with specificities of 83.3%, 88.8%, and 80.6%. The area under the receiver operating characteristic (ROC) curve (AUC) for the model constructed using these RNAs was 0.92, demonstrating its high diagnostic accuracy, particularly in distinguishing stage 0 BC from cancer-free individuals. Additionally, this model significantly outperformed traditional biomarkers such as CEA and CA15-3.^48^ Wang et al. identified six plasma exosomal tRFs—tRF-Glu-CTC-003, tRF-Gly–CCC–007, tRF-Gly–CCC–008, tRF-Leu-CAA-003, tRF-Ser-TGA-001, and tRF-Ser-TGA-002—that were downregulated in early breast cancer (EBC) patients compared to healthy controls. The combined AUC for these six tRFs was 0.844, with a sensitivity of 87.5% and specificity of 68.8%, suggesting superior diagnostic performance compared to individual tRFs.^18^

### Exosomal tRFs in colorectal cancer

Lu et al. demonstrated that tRF-3 was the most abundant tRF subtype in tissue and plasma exosomes of colorectal cancer (CRC) patients. The expression of three tRFs—tRF-3022b, tRF-3030b, and tRF-5008b—was significantly elevated in CRC patients compared to healthy controls. The combined AUC for these three tRFs was 0.8684, indicating their superior diagnostic potential compared to traditional biomarkers like CEA and CA19-9y.^39^^,^^49^

### Exosomal tRFs in pancreatic cancer

Chen et al. found that serum exosomal tRF-GluCTC-0005 was significantly elevated in PDAC patients with early liver metastasis. Higher levels of tRF-GluCTC-0005 correlated with larger and deeper metastatic tumors, suggesting that this tRF could be a potential biomarker for detecting liver metastasis in PDAC. Moreover, tRF-GluCTC-0005 showed higher sensitivity and specificity than traditional diagnostic marker CA19-9.^36^

Cao et al. reported that tRF-19-PNR8YPJZ was highly expressed in pancreatic cancer (PC) tissue samples and that exosomal tRF-19-PNR8YPJZ derived from pancreatic stellate cells (PSCs) promoted tumor proliferation and migration by regulating AXIN2. This tRF was associated with poor clinical outcomes, and its diagnostic potential was validated with an AUC of 0.8388.^42^

### Exosomal tRFs in non-small cell lung cancer

Peng et al. found that i-tRF-AspGTC and tRF-1-SerCGA were significantly downregulated in serum exosomes from Non-Small Cell Lung Cancer (NSCLC) patients compared to healthy controls. The combination of these tRFs with traditional biomarkers such as CEA and CYFRA21-1 significantly improved the diagnostic accuracy for NSCLC.^50^

Similarly, Zheng et al. demonstrated that several exosomal tRFs, including tRF-Leu-TAA-005, tRF-Asn-GTT-010, tRF-Ala-AGC-036, tRF-Lys-CTT-049 and tRF-Trp-CCA-057 were downregulated in NSCLC patients, with combined AUC values indicating their potential as diagnostic biomarkers.^20^

### Exosomal tRFs in endometrial cancer

Qian et al. identified that serum exosomal tRF-20-S998LO9D was significantly downregulated in endometrial cancer (EC) patients. The ROC curve for tRF-20-S998LO9D showed an AUC of 0.768, suggesting that it could serve as a novel biomarker for EC diagnosis.^43^

To establish the clinical utility of exosome-associated tRF biomarkers, further research is required, including larger clinical cohorts and more robust data collection. While exosome-associated tRFs hold significant promise, integrating them with traditional biomarkers such as CEA and CA19–9 may enhance diagnostic accuracy. With the accumulation of more data from multi-center collaborations and clinical trials, exosome-associated tRFs are expected to become valuable tools in cancer diagnostics and prognostics.

In light of the clinical potential of tRFs, various clinical trials are underway to evaluate tRFs as biomarkers for cancer and other diseases (Table 2). These trials, though in early phases, hold the promise that tRFs will eventually become widely adopted as reliable biomarkers in clinical practice. Table 1 has summarized the clinical trial data of tRFs as biomarkers for disease diagnosis or prognosis at home and abroad (data from https://www.chictr.org.cn/ and https://clinicaltrials.gov/). Furthermore, as an emerging clinical biomarker, the majority of ongoing clinical trials are currently in phase 0, with and some projects have not yet started to recruit subjects. Nevertheless, we firmly believe that with the support of more and more research data, tRFs will become highly promising and dependable tumor biomarkers, and eventually move to the clinic.

**Table 2:** Ongoing clinical trials involving tsRNA as a biomarker and diagnostic study for various diseases.

Registration number/NCT number	Study type	Study phase	Recruiting status	Disease	Sample name	Sample size
ChiCTR2400090283	Observational study	N/A	Not yet recruiting	Diabetes mellitus with ischemic stroke	Blood	120
ChiCTR1900022806	Basic science	0	Not yet recruiting	Sepsis	Blood	100
ChiCTR2000031507	Diagnostic test	0	Recruiting	Esophageal squamous cell carcinoma	blood, saliva, esophageal squamous cell carcinoma	1346
ChiCTR2300078534	Observational study	N/A	Recruiting	Intrahepatic cholestasis of pregnancy	Serum	60

## Conclusions and prospects

Exosome-associated tRFs have emerged as a significant focus in cancer research due to their involvement in tumor progression and their potential clinical applications. These small RNA fragments regulate gene expression and signal transduction through exosome-mediated intercellular communication, influencing key processes such as tumor cell proliferation, metastasis, immune evasion, and drug resistance. The stability and cancer-specific expression patterns of exosome-associated tRFs position them as promising biomarkers for early cancer detection, monitoring therapeutic response, and evaluating prognosis. Moreover, therapeutic strategies targeting specific tRFs have shown encouraging potential in modulating tumor progression and overcoming treatment resistance. Thus, exosome-associated tRFs represent a valuable area of exploration for advancing cancer diagnostics and treatment, with the potential to improve patient outcomes and disease management.

Despite these promising developments, there are several critical challenges that must be addressed to fully harness the clinical utility of exosome-associated tRFs. First, it is essential to develop standardized and efficient methods for exosome isolation and tRF detection to improve the reproducibility and comparability of research results across different studies. Currently, variations in isolation techniques and analytical methods may contribute to inconsistencies in findings. Second, more in-depth investigations are needed to elucidate the precise functional roles and mechanisms of exosome-associated tRFs in different cancer types and at various disease stages. Such studies will provide a more comprehensive understanding of how tRFs influence tumor biology and could identify cancer-specific tRF signatures with diagnostic and therapeutic relevance.

Additionally, while preclinical research has demonstrated the potential of exosome-associated tRFs, further validation in clinical settings is required to substantiate their efficacy and safety as diagnostic biomarkers and therapeutic targets. Large-scale clinical trials will be necessary to confirm their clinical applicability and to identify the most effective strategies for incorporating tRFs into personalized cancer therapy. Furthermore, the integration of multidisciplinary approaches and cutting-edge technologies, such as single-cell sequencing, high-throughput screening, and advanced bioinformatics tools, will accelerate the discovery and application of exosome-associated tRFs. These technologies can provide insights into the cellular and molecular heterogeneity of cancer, helping to tailor treatments to individual patients and improve therapeutic outcomes.

Looking forward, the continued research into exosome-associated tRFs holds the promise of revolutionizing cancer diagnosis and treatment. With ongoing advancements in technology and collaborative research efforts, these small RNA molecules have the potential to serve as critical biomarkers and therapeutic targets in personalized oncology. As the field progresses, the use of exosome-associated tRFs could significantly enhance the accuracy of early cancer diagnosis, optimize treatment strategies, and ultimately improve survival rates and quality of life for cancer patients.

In conclusion, the growing body of evidence underscores the clinical potential of exosome-associated tRFs as novel biomarkers and therapeutic targets. Their integration into cancer management could represent a significant breakthrough in the fight against cancer, paving the way for more effective and individualized treatment approaches.

## CRediT authorship contribution statement

**Yanru Pan:** Writing – review & editing. **Bo Zhang:** Writing – review & editing. **Zhe Li:** Writing – review & editing. **Kefeng Hu:** Writing – review & editing, Funding acquisition.

## Funding

This study was supported by the grants from grants from Ningbo Top Medical and Health Research Program (2023020612), the Ningbo Leading Medical & Healthy Discipline (2022-S04) and the Medical and Health Research Project of Zhejiang Province (2023RC262).

## Conflict of interests

The authors declare that they have no conflict of interests.
